# THz Acoustic Spectroscopy by using Double Quantum Wells and Ultrafast Optical Spectroscopy

**DOI:** 10.1038/srep28577

**Published:** 2016-06-27

**Authors:** Fan Jun Wei, Yu-Hsiang Yeh, Jinn-Kong Sheu, Kung-Hsuan Lin

**Affiliations:** 1 Institute of Physics, Academia Sinica, Taipei 11529, Taiwan; 2 Department of Photonics, National Cheng Kung University, Tainan 70101, Taiwan; 3 Institute of Photonics Technologies, National Tsing Hua University, Hsinchu 30013, Taiwan

## Abstract

GaN is a pivotal material for acoustic transducers and acoustic spectroscopy in the THz regime, but its THz phonon properties have not been experimentally and comprehensively studied. In this report, we demonstrate how to use double quantum wells as a THz acoustic transducer for measuring generated acoustic phonons and deriving a broadband acoustic spectrum with continuous frequencies. We experimentally investigated the sub-THz frequency dependence of acoustic attenuation (i.e., phonon mean-free paths) in GaN, in addition to its physical origins such as anharmonic scattering, defect scattering, and boundary scattering. A new upper limit of attenuation caused by anharmonic scattering, which is lower than previously reported values, was obtained. Our results should be noteworthy for THz acoustic spectroscopy and for gaining a fundamental understanding of heat conduction.

Acoustic properties in the THz regime are fundamental to the understanding of materials. For example, the origin of the Boson peak at ~1 THz (caused by excessive vibrational states) in glass and amorphous materials has been puzzling for several decades^1^,^2^. The breakdown of Fourier’s law, which is used for modeling heat conduction, has also attracted considerable attention recently. When ballistic thermal conduction in SiGe was found found to be unexpectedly long (on the micron scale), sub-THz phonons were considered to play an essential role^3^,^4^. The frequency dependence of phonon mean-free paths (MFPs) is vital to understanding the underlying physics of heat conduction. However, frequency-dependent phonon MFPs still cannot be directly resolved, although a few techniques have been demonstrated for experiments on the relation between phonon MFPs and thermal conductivity^5^,^6^,^7^,^8^.

Typically, THz incoherent phonons in materials have been studied by Raman^9^, neutron^10^, and X-ray scattering^11^, but the phase information cannot be obtained by these techniques. Picosecond ultrasonics^12^ has been widely used to study the interaction between coherent phonons and materials, but the frequencies involved are typically limited to below 500 GHz^13^. THz coherent acoustic phonons in semiconductor heterostructures have been studied^14^,^15^, and the signals in GaN-based materials have been determined to be strong because of piezoelectric effects^14^,^15^,^16^,^17^. The acoustic frequency of this technique can be up to 2.5 THz^18^,^19^. Applications such as waveform synthesis^20^,^21^, acoustic spot modulation^22^, nondestructive images^23^, and phononic device characterization^24^ have been demonstrated. Recently, this technique has been used to study fundamental problems in materials such as GaN^25^, silica^2^,^26^, ice^27^, and water^28^.

When a single quantum well (QW) is used as an acoustic transducer^24^,^26^,^27^,^28^, an acoustic signal initiated in the single QW is completely overwhelmed by strong transient electronic signals near zero time delay. However, it is necessary to measure the acoustic signals from a transducer in numerous cases of acoustic analysis. For multiple QWs^2^,^14^,^15^,^18^,^19^,^20^,^22^,^23^,^25^,^29^,^30^,^31^,^32^, the frequency components are limited because of the spatial period of the QWs, although obtaining approximations of the generation signals is feasible. Therefore, the frequency dependence of phonon MFPs in the sub-THz regime has not been experimentally and comprehensively investigated, despite the importance of the THz phonon properties of acoustic transducers.

Previous research has been stymied by the problem that an initiated acoustic signal is completely overwhelmed by the strong transient electronic signals near zero time delay; in this report, we explain how to circumvent this problem. We demonstrate how to use double QWs as a THz acoustic transducer for measuring the generated acoustic phonons and the broadband acoustic spectra with continuous frequencies from a single trace measurement. We investigated the frequency dependence of acoustic attenuation in GaN in the THz regime. In contrast to previous reports^25^,^29^,^30^, in which one effective MFP for all phonon frequencies or phonon MFPs for a few frequencies have been measured, we experimentally obtained MFPs of continuous frequencies in GaN within THz frequencies and analyzed relevant mechanisms such as anharmonic decay, defect scattering, and boundary scattering. Our demonstration can characterize the acoustic properties of the transducer, which is crucial to THz acoustic spectroscopy. This technique can also be applied to measure frequency-dependent phonon MFPs for studies of heat conduction^3^,^4^,^5^,^6^,^7^,^8^ or the Boson peak in glass^1^,^11^.

## Principles

**Figure 1:**
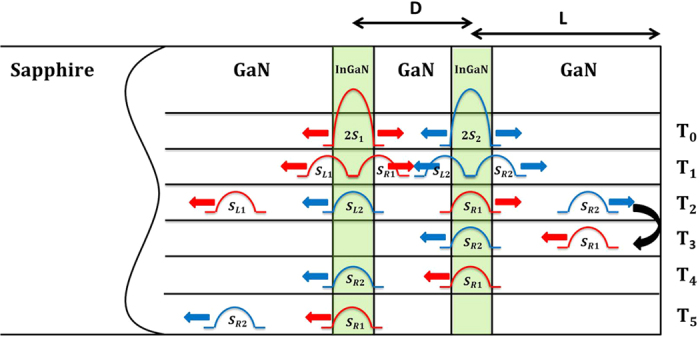
The schematic of propagating acoustic pulses in the transducer with structure of double quantum wells. After photoexcitation at T_o_, the four acoustic pulses propagate from the time T_1_ to time T_5_.

Figure 1 illustrates a schematic of a THz acoustic transducer. The band gap of InGaN QWs is lower than that of GaN QWs. After the femtosecond optical pulses, possessing appropriate photon energy, excite carriers only in the QWs, acoustic pulses can be initiated in each QW and propagate in opposite directions^18^,^22^. Because the absorption coefficient of the QWs can be modulated by strain pulses, the duration of acoustic pulses traveling through the same QWs can be detected by measuring the transmission of the optical pulses^18^. Therefore, the generation and detection of acoustic pulses can be achieved using a GaN-based structure and pump-probe techniques. THz acoustic spectroscopy of other materials, on top of the GaN cap layer, can also be investigated.

After the acoustic pulses are generated in the QWs at time T_0_ (Fig. 1), the pulses propagate out from the QW regions in opposite directions. The temporal response of the transmitted optical probe pulses can be modeled as^17^,^18^


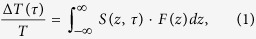


where *S*(*z, τ*) is the time evolution of the longitudinal strain distribution, and *F*(*z*) is the sensitivity function of the acoustic transducer. In the frequency domain, the optical signals resulting from the generated acoustic pulses are^13^


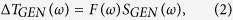


where 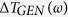 is the Fourier transform of the experimental data for acoustic generation, 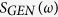 is the acoustic spectrum immediately after photoexcitation, and *F*(*ω*) can be derived from *F*(*z*)^13^ in Eq. (1). After the strain pulses propagate toward the GaN surface, as shown in Fig. 1, the optical signals associated with the echoed pulses are


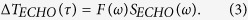


Moreover, the echoed strain pulses are


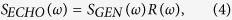


where *R*(*ω*) is the response function of the material that characterizes mechanisms such as attenuation, dispersion, and scattering. According to Eqs (2)–(4),13[Disp-formula eq10]


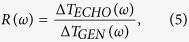


which can be obtained from the experimental data without sensitivity functions.

In previous studies, acoustic analyses for multiple QWs entailed using only one function *S*(*z, τ*) for generating phonons or echoed phonons. However, the acoustic signals, from τ  = T_0_ to τ = T_1_ (Fig. 1), tended to be experimentally overwhelmed by the strong electronic signals near zero time delay and were not resolvable. These signals were actually ignored in previous acoustic analyses for multiple QWs^2^,^14^,^15^,^18^,^19^,^20^,^22^,^23^,^25^,^31^,^32^,^33^,^34^. Moreover, such signal from single QW was completely nonmeasurable^21^,^24^,^26^,^27^,^28^. To circumvent this problem, we divide the functions *S*(*z, τ*) in Eq. (1) for realizing acoustic analysis of double QWs. Because the strain functions are associated with the wave functions of the QWs^17^,^18^, one can reasonably assume that the generating strain pulses from each QW can be effectively separated in our experimental condition.

In Fig. 1, the strain functions *S*_*i*_ are used for both QWs, where *i* = 1, 2. After photoexcitation, only the propagating part of the strain (or strain pulses) is considered. For lossless media, the propagating strain pulses *S*_*i*_(*z, τ*) can be represented by *S*_*i*_(*z* ± *vτ*), where *v* denotes the longitudinal acoustic velocity of GaN. Notably, the difference in acoustic velocity and acoustic impedance between the In_0.14_Ga_0.86_N QW and GaN are neglected. We further assume that the acoustic attenuations of 3-nm-thick QWs are negligible, and that the acoustic waveform remains the same for the period in which the acoustic pulses cross the QW region.

Immediately after T_0_ in Fig. 1, we consider four strain pulses with two counter-propagating directions, *S*_*L*1_(*z, τ*), *S*_*R*1_(*z, τ*), *S*_*L*2_(*z, τ*), *S*_*R*2_(*z, τ*). The optical responses at T_2_ and T_4_ are


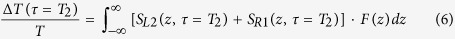


and


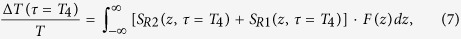


respectively. Notably, *S*_*i*_(*z, τ*) varies as a function of *τ*. At T_0_ in Fig. 1, the propagating strain distribution of a QW *i* can be represented as


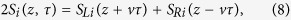


where 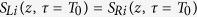
17. Assume that the strain functions have time-reversal symmetry^17^; 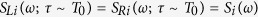 in the period in which the initiated pulses depart from the QWs at nearly T_0_ or T_1_ (Fig. 1). Subsequently, at nearly T_2_, the Fourier transform of the optical response is


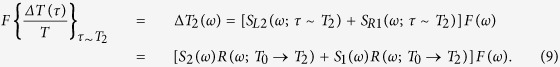


Therefore, the optical responses at nearly T_2_ and T_4_ can be derived as


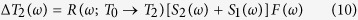


and


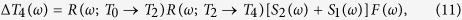


respectively. From Eq. (10), the information of the generated pulses can be experimentally obtained. 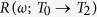 is typically dominated by the attenuation of GaN; that is, 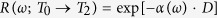, where *α*(ω) is the attenuation in GaN and *D* is the distance between the QWs in Fig. 1. The frequency-dependent attenuation of GaN is also included in 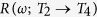 in Eq. (11). According to Eqs (10) and (11), one can derive


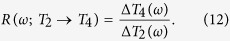


In the following section, *R*(ω) represents the acoustic response function from T_2_ to T_4_ in Fig. 1. We also discuss the mechanisms contributing to *R*(ω) such as acoustic attenuation and surface scattering.

## Results and Discussions

**Figure 2:**
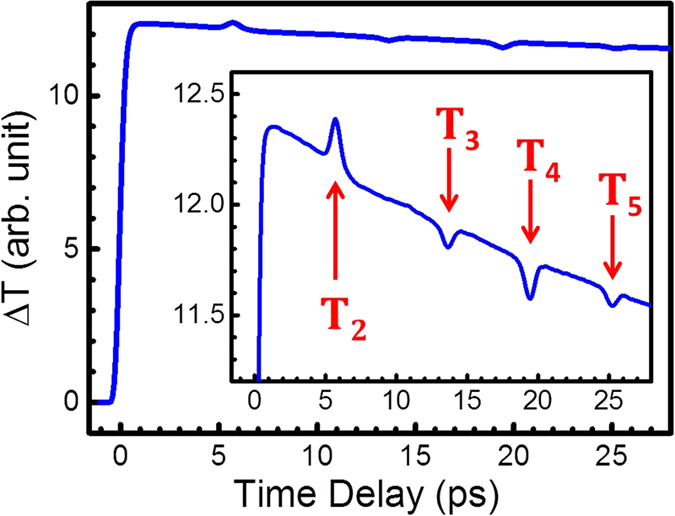
The transmission changes of the optical probe pulses as a function of time delay between the pump and probe. The optical signals, caused by the acoustic pulses crossing the quantum wells as illustrated in Fig. 1, are highlighted in the inset.

Figure 2 presents the optical transmission changes as a function of time delay between the pump and probe pulses. The sample was measured at room temperature. A strong rising signal was measured at approximately zero time delay, followed by a slow relaxation, resulting from the band-filling effects in the QWs. The inset of Fig. 2 highlights the pulse-like features caused by the strain pulses crossing the QWs. The pulses are labeled with times corresponding to the schematic in Fig. 1. As mentioned in the Principles section, the acoustic signals at nearly T_0_ were overwhelmed by the strong electronic signals. Note that the signs of the acoustic signals were reversed after acoustic reflection at the free-end surface of GaN.

**Figure 3:**
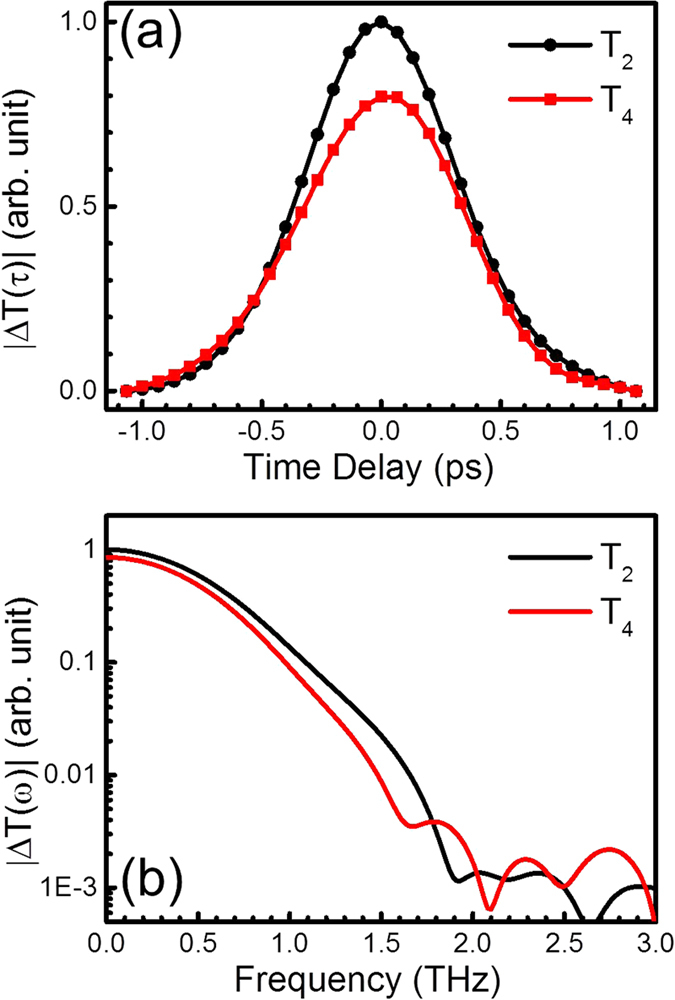
(**a**) The extracted signals associated with the acoustic pulses crossing the quantum wells at T_2_ and T_4_ in Fig. 2 and (**b**) their corresponding spectra.

Figure 3(a) illustrates the optical signals induced by the acoustic pulses at nearly T_2_ and T_4_, which were extracted through background subtraction from the trace in Fig. 2. The time axes of the pulses were shifted and the signals were taken as absolute values for easier comparison. The corresponding Fourier spectra, 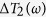 and 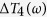, are shown in Fig. 3(b). The response function *R*(ω) can thus be obtained according to Eq. (8).

The signals in Fig. 3(b) are plotted up to an acoustic frequency of 3 THz; however, determining the valid range of acoustic frequencies for analysis is imperative. The frequency response is determined by multiple factors such as the acoustic pulse shape, sensitivity function, and optical probe pulses used for detection^17^,^18^,^22^. If the sensitivity functions and optical pulse shape can be precisely determined, there is no frequency limit for detection with deconvolution. Nevertheless, the valid frequency range was practically dominated by noise levels in the experimental data. According to Fig. 3(b), signals above 1.5 THz might lie at the noise level, which is comparable to the report of a previous study^26^. We only investigated the response function within 1 THz in a conservative manner.

To analyze the mechanisms of *R*(*ω*) in GaN, monitoring, for example, *S*_*R*1_, from T_2_ to T_4_ in Fig. 1 is required. In addition to acoustic attenuation in GaN, the acoustic scattering at the GaN/air interface should be considered^33^. The acoustic response engendered by the interface can be separated into two parts, with and without frequency dependence. Notably, the acoustic dispersion of GaN is linear at least up to 4 THz. The acoustic transmission or reflection at the interface, resulting from the acoustic mismatching of two different materials, does not possess frequency dependence under our experimental conditions. However, the specular scattering probability of acoustic phonons at the interface is frequency-dependent^33^.

**Figure 4:**
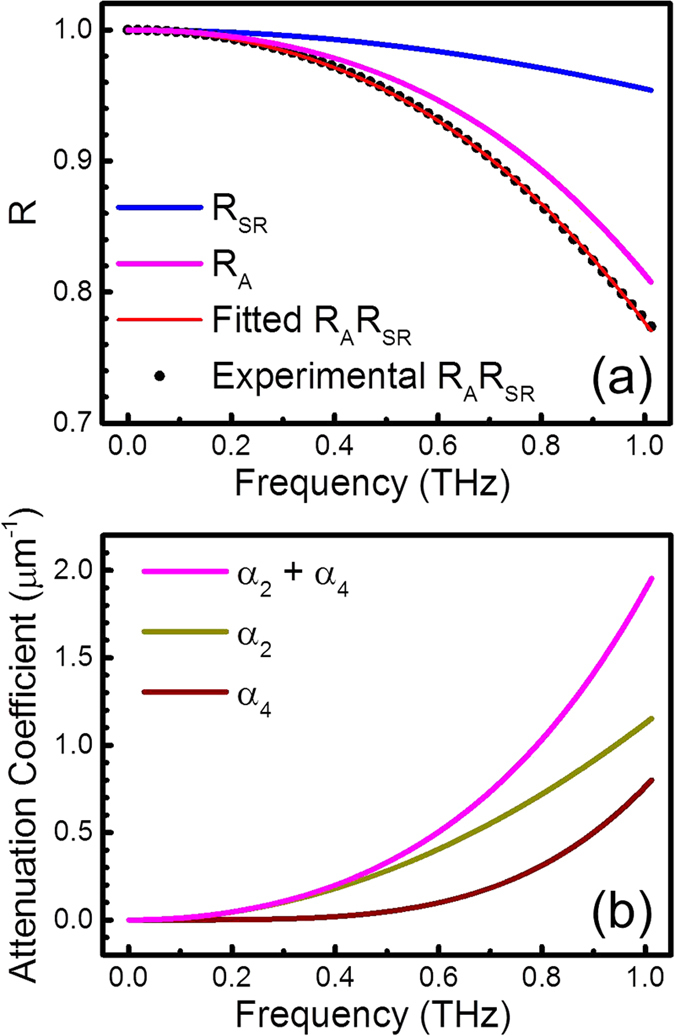
(**a**) The experimentally obtained acoustic response function and the fitting curves resulting from acoustic attenuation, surface roughness, and both of them. (**b**) The fitted attenuation coefficients of GaN *α*(*ω*), *α*_2_(*ω*) and *α*_4_(*ω*).

Because we were interested in the frequency-dependent part of *R*(*ω*), the experimentally obtained response function was normalized as shown by the black dots in Fig. 4(a). We considered the acoustic attenuation *R*_*A*_(*ω*) in GaN and the specular scattering probability from the GaN surface *R*_*SR*_(*ω*). To distinguish these two contributions, we measured the surface roughness of the GaN with an atomic force microscope. The root mean square (rms) of the surface height was 0.193 nm, indicating the surface is atomically flat. *R*_*SR*_(*ω*) can be theoretically calculated according to the model of small slope approximation^33^,^35^.


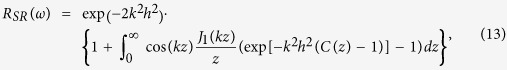


where *h* is the rms of the surface height, *k* = *ω/v* is the wave number, *J*_1_ is the Bessel function of the first kind, and *C*(*z*) is the surface correlation function. The blue curve in Fig. 4(a) shows the calculated *R*_*SR*_(*ω*) according to our experimental condition, *h* = 0.193 nm.

The mechanisms of acoustic attenuation considered here include anharmonic scattering, line defect scattering, and point defect scattering^36^. Anharmonic scattering results from the intrinsic properties of perfectly crystalline GaN and exhibits a frequency dependence with a power of 2. The attenuation resulting from line defects, which are common in GaN thin films on sapphire substrates^37^, also demonstrates a frequency dependence with a power of 2. The attenuation resulting from point defects, such as nitrogen vacancies and donor impurities in GaN^38^, exhibits a frequency dependence with a power of 4. The attenuation coefficient is


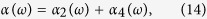


where 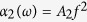 and 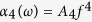 with *ω* = 2*πf*. The response function associated with attenuation is


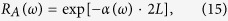


where 2*L* = 110 nm is the propagation length in GaN from T_2_ to T_4_ in Fig. 1. We fitted the experimental results *R*_*A*_(*ω*)*R*_*SR*_(*ω*), as shown by the red curve in Fig. 4(a), with the parameters *A*_2_ and *A*_4_. Figure 4(b) shows the fitted attenuation coefficients *α*(*ω*), *α*_2_(*ω*) and *α*_4_(*ω*) of GaN at room temperature. The parameters *A*_2_ and *A*_4_ are listed in Table 1 in bold type.

Note that *A*_2_ includes contributions from both anharmonic scattering and line defect scattering, but anharmonic scattering is temperature-dependent, whereas line defect scattering is not. We can thus distinguish the contributions with the relation 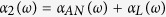, where *α*_*AN*_(*ω*) and *α*_*L*_ (*ω*) denote the anharmonic scattering and line defect scattering, respectively. The mechanism of anharmonic scattering follows the relation^25^,^36^


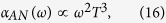


where *T* represents the temperature. As the temperature declined, 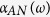 decreased, whereas 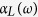 remained constant. Theoretically, 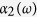 should also decrease with the temperature, and the percentage of the contribution can be extracted from 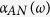 to 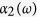 by fitting with an additional parameter *T*.

We conducted another set of temperature-dependent experiments. After the aforementioned analysis for each temperature, the parameters *A*_2_ and *A*_4_ were obtained, as listed in Table 1 in black. However, 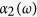 did not monotonically decrease with the temperature. These results indicate that 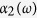 should be dominated by temperature-independent defect scattering. Furthermore, the number of defects is not homogeneous in our GaN sample. The focused laser spot, which had a diameter on the order of 20 μm, did not remain on the same area of the sample for each temperature. Notably, as the temperature varied, thermal expansion caused the sample on the cold finger in the cryostat to shift.

We could still determine the upper limit of 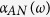 from our experimental results, although we could not exactly obtain the intrinsic property of anharmonic scattering in GaN. Because 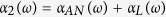, the extreme case of a perfect crystal occurs when 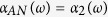 and 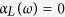. By calibrating the temperature effect of anharmonic scattering 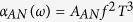 in Table 1, we observed that the measured GaN region at a temperature of 260 K demonstrated the minimum attenuation caused by defect scattering; consequently, the upper limit for *A*_*AN*_ was 3.24 × 10^−8^(*ps*)^2^(*μm*)^−1^*K*^−3^.

Reference 25 reported that the anharmonic decay rate 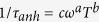, where *c* = (6 ± 0.2) × 10^−23^, *a* = 1.98 ± 0.27, and b is set as 3.08. If the longitudinal acoustic velocity *v* = 8 nm/ps is used^39^, the corresponding *A*_*AN*_ from ref. 25 is approximately 8 times higher than the value we obtained. We argue that anharmonic scattering of GaN was overestimated in ref. 25; this argument is supported by the claim in ref. 30 that the intrinsic attenuation coefficient of GaN for 45 GHz is significantly smaller than the measured value of 65.8 cm^−1^ at room temperature (295 K). According to our experimental results, *α*_*AN*_ for 45 GHz should be lower than 16.8 cm^−1^, which agrees with the reports in ref. 30. However, ref. 25 overestimated *α*_*AN*_ at 45 GHz as 143 cm^−1^. Notably, according to our experimental results, the actual value of *A*_*AN*_ could be even lower.

## Conclusions

We demonstrate how to use two QWs as a THz acoustic transducer for measuring generated acoustic phonons and obtaining a broadband acoustic spectrum with continuous frequencies. This method circumvents the problems of previous designs and enables experimentally investigating the sub-THz frequency dependence of phonon MFPs in GaN. We observed that the acoustic attenuation in our GaN sample was dominated by defect scattering. The anharmonic scattering of GaN, which reflects the intrinsic attenuation of a perfect crystal, was quantitatively analyzed. The upper limit of acoustic attenuation, caused by anharmonic scattering, was obtained and the actual attenuation of GaN could be even lower.

## Methods

### Sample Design

A c-plane hexagonal GaN buffer layer, with a thickness on the order of 2 μm, was grown on a double-polished c-plane sapphire substrate through metal-organic chemical vapor deposition. The structure on the top of the unintentionally doped GaN buffer layer is illustrated in Fig. 1. D was approximately 46 nm and L was approximately 55 nm. The thickness of each In_0.14_GaN QW was 3 nm. The peak of photoluminescence from the QWs was located at approximately 2.9 eV. The roughness of the GaN surface was 0.193 nm, as derived from an atomic force microscope measurement. The density of dislocation defects was estimated to be on the order of 4 × 10^8^ cm^−2^, according to etch pit density measurement.

### Experimental Setup

The sample was mounted on a holder in a cryostat. The pressure of the chamber was lowered to below 10^−4^ mTorr for temperature-dependent measurements. Typical degenerate and noncollinear pump-probe measurements were conducted at 390 nm. The polarization of the pump beam was orthogonal to that of the probe beam. The repetition rate of the pulses was 80 MHz. The diameter of the optical spots on the sample was measured to be approximately 22 μm with a 10 μm pinhole. The pump and probe fluences were approximately 184 and 30 μJ/cm^2^, respectively. A polarizer was placed in front of the photodetector to eliminate pump light leakage. The pump beam was modulated at 677 kHz with an acousto-optical modulator. A lock-in amplifier was used to record the transmission variation of the probe pulse as a function of time delay between the pump and probe pulses.

## Additional Information

**How to cite this article**: Wei, F. J. *et al*. THz Acoustic Spectroscopy by using Double Quantum Wells and Ultrafast Optical Spectroscopy. *Sci. Rep.* **6**, 28577; doi: 10.1038/srep28577 (2016).

## Figures and Tables

**Table 1:** The fitted coefficients *A*_2_ and *A*_4_, as mentioned in the text, for each temperature.

Temperature	*A*_2_[(*ps*)^2^(*μm*)^−1^]	A_4_[(*ps*)^4^(*μm*)^−1^]
300 K	**1.13**	**0.76**
300 K	1.25	1.05
280 K	0.79	0.91
260 K	0.57	1.23
240 K	0.56	0.81
220 K	0.78	0.76
200 K	1.16	0.65
180 K	0.87	1.07
160 K	0.95	1.14

The values in bold type come from different set of measurements.
